# Liver Resection for Primary Hepatic Angiosarcoma: Bicentric Analysis of a Challenging Entity

**DOI:** 10.3390/jcm11112990

**Published:** 2022-05-25

**Authors:** Shadi Katou, Claudine Di Pietro Martinelli, Carolina Silveira, Franziska Schmid, Felix Becker, Sonia Radunz, Mazen Juratli, Haluk Morgul, Vanessa Banz, Andreas Pascher, Andreas Andreou, Benjamin Struecker

**Affiliations:** 1 Department for General, Visceral, and Transplant Surgery, University Hospital Muenster, 48149 Muenster, Germany; c_silv01@uni-muenster.de (C.S.); franziska_schmid96@yahoo.de (F.S.); felix.becker@ukmuenster.de (F.B.); sonia.raduenz@ukmuenster.de (S.R.); mazen.juratli@ukmuenster.de (M.J.); haluk.morguel@ukmuenster.de (H.M.); andreas.pascher@ukmuenster.de (A.P.); benjamin.struecker@ukmuenster.de (B.S.); 2 Department of Visceral Surgery und Medicine, Inselspital, Bern University Hospital, University of Bern, 3010 Bern, Switzerland; claudine.dipietromartinelli@insel.ch (C.D.P.M.); vanessa.banz@insel.ch (V.B.); andreas.andreou@insel.ch (A.A.)

**Keywords:** hepatic angiosarcoma, hepatic resection, overall survival, tumor rupture

## Abstract

Primary hepatic angiosarcoma (PHA) is a rare malignant tumor of the liver, and data on patient outcome after surgical treatment are scarce. The aim of this study was to evaluate postoperative morbidity and overall survival (OS) of patients who underwent hepatectomy for PHA. This is a bicentric retrospective analysis of all consecutive patients who underwent liver resection in curative intent for PHA between 2012 and 2019 at the University Hospital of Muenster and the University Hospital of Bern. Nine patients (five female, four male) were included from both centers. Median age was 72 years (44–82). Most lesions (77.8%) were larger than 5 cm, and mean size of the biggest lesion was 9.4 ± 4.5 cm. Major hepatectomy was performed in four (44.4%), and radical resection (R0) was achieved in six (66.7%) patients. Postoperative complication rate was 88.8%, including 44.4% higher than 3a in the Clavien–Dindo classification. OS survival rates at 1, 2, and 3 years were 44.4%, 22.2%, and 12.5%, respectively, and median OS was 5 months. OS was significantly better after radical resection (R0: 15 months vs. R1: 0 months, *p* = 0.04), whereas presentation with tumor rupture at diagnosis was associated with the worst OS (0 months vs. 15 months, *p* = 0.005). Disease recurrence occurred in three patients (33.3%) between three and seven months after surgery. Radical resection remains the only potentially curative treatment option for PHA. However, postoperative morbidity is high, and the overall prognosis remains poor. Multimodal therapy options and management strategies are urgently needed and could improve the prognosis of patients suffering from PHA in the future.

## 1. Introduction

Primary hepatic angiosarcoma (PHA) is a very rare entity, as it barely represents 5% of all angiosarcomas and less than 2% of all primary hepatic malignancies. However, PHA is a highly aggressive disease associated with a poor prognosis [1,2,3,4,5]. Given the unspecific symptoms of this disease, such as abdominal pain and fatigue, the diagnosis of PHA is challenging and often made at an advanced stage, when multiple lesions or even metastases are already present [6,7]. In other cases, diagnosis follows tumor rupture with hemoperitoneum, which can result in death, and must therefore be managed urgently [8,9]. The reliability of radiologic examinations is limited with regard to PHA diagnosis, and therefore, in most cases, a lesion biopsy is required. Frequently, histological diagnosis of PHA is confirmed after resection of the tumor [10]. Exposure to vinyl chloride monomer (VCM), inorganic arsenic, thorotrast, and the use of androgenic steroids are described as risk factors for PHA, but the main etiology remains unclear [11,12]. Since PHA is rare, there is a scarcity of data on the best therapy and prognosis of this disease. Radical resection is currently considered the only effective treatment option [13,14,15]. Therefore, the aim of this study was to evaluate the postoperative outcomes, overall survival (OS), and disease-free survival (DFS) after liver resection for PHA.

## 2. Materials and Methods

### 2.1. Study Design and Inclusion Criteria

This study was conducted at the University Hospital of Muenster, Germany, and at Inselspital in Bern, Switzerland, both tertiary centers for hepatobiliary (HPB) surgery. The study was approved by the local ethics committees at the University of Muenster (ID 2019-636-f-S) and the University of Bern (ID 2021-00333). We performed a retrospective analysis of all patients who underwent liver resection for PHA between 2012 and 2019. Patients < 18 years old were excluded from the study.

### 2.2. Preoperative Assessment

All patients in this study underwent standard preoperative evaluation. This included medical history, physical examination, laboratory tests, imaging studies, and anesthesia evaluation. Cross-sectional imaging, including contrast-enhanced computed tomography, magnetic resonance imaging, and ultrasound, were used to determine the location and extent of the lesion and to evaluate the tumor entity. If necessary, percutaneous biopsy of the lesion was performed. All elective patients were presented and discussed at the local interdisciplinary tumor board, consisting of HPB surgeons, hepatologists, oncologists, specialized radiologists, and pathologists. An individualized treatment plan was recommended for each patient.

### 2.3. Surgical Procedure and Postoperative Management

Liver resections were either atypical or anatomical hepatectomies. Depending on tumor size and feasibility of resection, procedures were performed either in laparoscopic or open technique. Following laparotomy or diagnostic laparoscopy, previously undiagnosed tumor spread or infiltration into surrounding organs was ruled out. Major liver resection was defined as resection of three or more liver segments. In case of major hepatectomy, an abdominal drain was placed. All patients were admitted to an intensive care or intermediate care unit after surgery, and were closely monitored for early postoperative complications. Abdominal drains were removed within the first three postoperative days if bile leakage was not detected. Postoperative morbidity was defined as any complication within 90 days after surgery, and was graded according to Clavien–Dindo classification [16]. Major morbidity was defined as any complication > 3a grade, and postoperative mortality as grade 5. Postoperative mortality was assessed at 30 and 90 days after surgery, and during the entire follow-up time.

### 2.4. Histological Evaluation

Histological evaluation confirmed the diagnosis of PHA in all resected specimens by specialized pathologists in both local institutions. Notably, the standard for histological classification of PHA differed between the two institutes, since the definition of tumor category according to the TNM classification is not standardized. Nevertheless, R0 resection was always defined as microscopically surgical margins negative for malignant cells < 1 mm.

### 2.5. Statistical Analysis

Clinicopathological characteristics such as age, gender, comorbidities, tumor characteristics of PHA, type of surgical procedure, and postoperative course were assessed. Furthermore, follow-up data were collected at 1, 2, 3, and 5 years after surgery, if applicable. OS was calculated from the day of surgery to death or last follow-up. DFS was calculated from the day of surgery to first diagnosis of recurrence or last follow-up. Variables were provided with median and range or frequency, as appropriate. Fischer exact and unpaired Student’s *t*-tests were carried out for comparison of categorical and continuous parameters, as appropriate. Analysis of OS was obtained by using the Kaplan–Meier method. Log-rank test was performed for the univariate analysis of factors associated with OS. A *p*-value < 0.05 was considered statistically significant. For multivariate analysis, Cox regression analysis with backward elimination was performed. Statistical analysis was performed using SPSS (IBM, Version 28.0, Armonk, NY, USA).

## 3. Results

### 3.1. Patient and Tumor Characteristics

In total, 14 patients with histologically confirmed PHA were identified. Thereof, 11 (78.5%) patients underwent surgical therapy. In two (14.2%) patients, PHA was incidentally found after liver transplantation, therefore, these patients were excluded from the study. Thus, in total, nine patients from both centers were identified and included for statistical analysis.

Patient characteristics are summarized in Table 1. Median age of all nine patients was 72 years (44–82). Leading clinical presentation was either abdominal pain in the upper quadrants (*n* = 4, 44.4%) or hemorrhage due to tumor rupture (*n* = 2, 22.2%), whereas other patients had no symptoms, and the lesion was found incidentally on imaging for other purposes (*n* = 3, 33.3%). Solitary lesions were more frequent (*n* = 7, 77.8%), and most lesions were larger than 5 cm (*n* = 7, 77.8%). Lesion biopsy was performed in six patients, but only confirmed diagnosis of PHA in four (66.7%) patients. None of the patients received neoadjuvant therapy, whereas four patients (44.4%) received adjuvant treatment following surgery. R0 resection was achieved in six patients (66.7%). Grading was described only in four (44.4%) cases, all of which were graded G3. Lymph node dissection of the hepatoduodenal ligament was performed in five (55.6%) patients, however none of the patients had lymph node metastases.

### 3.2. Postoperative Morbidity and Mortality

Postoperative morbidity rate and major morbidity rate were 88.8% (*n* = 8) and 44.4% (*n* = 4), respectively. Thirty-day mortality rate was 22.2% (*n* = 2), where both patients presented with ruptured tumor and died on the second and ninth postoperative day due to liver and respiratory failure. It is worth mentioning that both patients undergoing emergency hepatectomy had an R1 resection. Ninety-day mortality rate was 44.4% (*n* = 4).

### 3.3. Oncologic Outcomes and Predictors for Overall Survival

**Figure 1:**
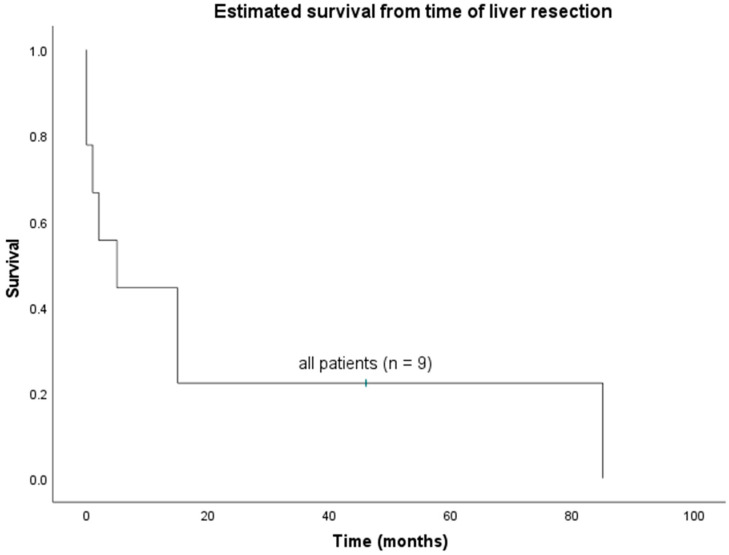
Overall survival of all patients undergoing hepatectomy for PHA (*n* = 9).

Median OS of all nine patients was 5 months (0–85 months) (Figure 1), whereas median OS and median DFS after R0 resection were 15 months (1–85 months) and 5.2 months (1–85 months), respectively. Only one (11.1%) out of nine patients is alive to date, 46 months after R0 resection, and with no signs of disease recurrence. The 1-, 2- and 3-year OS rates were 44.4%, 22.2%, and 12.5%, respectively. Disease recurrence was reported in three patients, at three, five, and seven months after surgery, and all patients died due to tumor progression. In two patients, the recurrence was within the liver; one patient had cerebral metastases. Another patient developed pulmonary metastases after liver resection and died. Post-mortem analysis, however, revealed that the metastases were from renal cell carcinoma, for which the patient had previously a curative treatment. In addition to patients with 30-day mortality, another patient died of multiple organ failure six weeks after surgery. The cause of death was unknown in one patient.

**Figure 2:**
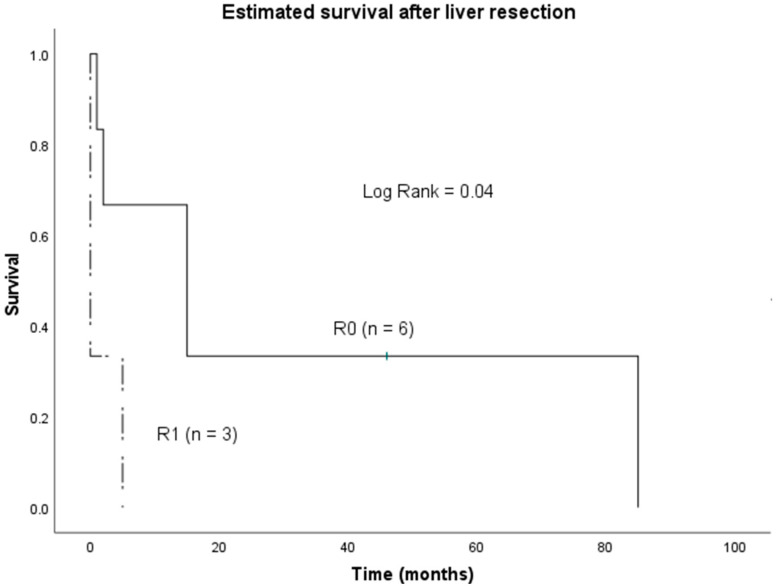
Overall survival of patients undergoing hepatectomy for PHA according to R status R0 vs. R1).

**Figure 3:**
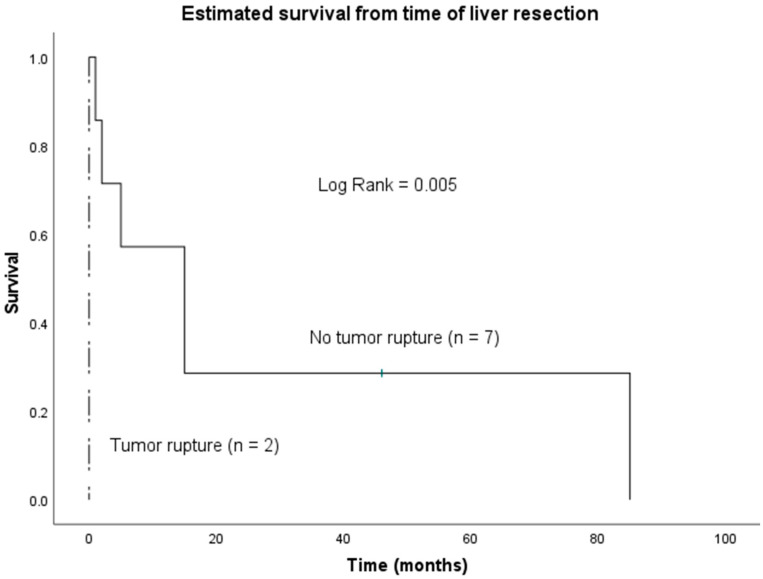
Overall survival of patients undergoing hepatectomy for PHA according to the presentation with or without tumor rupture.

In univariate analysis, R1 status (R0 vs. R1, *p* = 0.04, Figure 2) and tumor rupture (rupture vs. no rupture, *p* = 0.005, Figure 3) were significantly associated with worse OS. In multivariate analysis, none of the analyzed factors showed a significant impact on OS (Table 2).

## 4. Discussion

Angiosarcomas are highly aggressive lesions, and are particularly rare in the liver. Diagnosis of PHA remains challenging since clinical presentation of PHA and radiological findings are usually unspecific [6,7,17]. Furthermore, no specific tumor markers are available, so far. PHA is often diagnosed at a late stage, and patients may even be initially presented with tumor rupture and intra-abdominal bleeding. Data regarding the optimal treatment strategy are scarce, with only a few case reports and small case series reporting on the treatment strategy and outcome. Overall prognosis associated with PHA remains poor, and resection is so far the only potentially curative treatment option [18,19].

In this study, we evaluated surgically treated patients with PHA from two European hepatobiliary centers. After excluding patients not qualifying for surgery and patients having undergone liver transplantation, we included nine patients for further analysis. Median OS of all patients was only 5 months, which is in accordance with a meta-analysis of 25 articles including 64 patients published by Zheng et al. [13]. Our R0-resected patients showed a significantly longer median survival (15 months, 1–85), compared to R1 resection (0 months, 0–5). Another significant difference in median OS was noted in patients presenting with ruptured tumor (0 months) in comparison to those without (15 months, 1–85). However, in our small collective of nine patients, we found no independent predictors for long-time survival of resected patients in multivariate analysis. Interestingly, radical lymph node dissection did not have a beneficial impact on survival in our cohort.

In a recent, and one of the largest case series published, Tripke et al. described the course of nine patients with PHA and their outcome after surgical treatment [14]. Our cohort was comparable with regard to number and gender of patients, although the median age in our group was higher (62 vs. 72 years). The rate of R1 resections in their cohort was 11.1% (*n* = 1) and PHA was graded G3 in only one patient. In our study, R1 rate was 33.3% (*n* = 3) and grading was determined only in four cases, all of which were G3. This may explain why we found a lower OS for surgically resected PHA in comparison to Tripke et al., who reported a median OS of 18 months.

Another relevant aspect explaining our outcome in our series was the number of patients that presented with hemorrhagic shock (*n* = 2; 22%) due to ruptured PHA. Both patients presenting with tumor rupture deceased within a few days after surgery. The poor prognosis of patients with ruptured PHA has been reported before, and even after surgical and interventional salvage measures, most patients survived no longer than one month [9,15]. Further, in the setting of an emergency hepatectomy, the possibility of an R1 resection could be increased.

The poor median survival of only 5 months In our cohort is generally dissatisfying, and illustrates that our understanding of the disease and treatment strategies for PHA have not improved in the past decade. On the one hand, this could be due to the challenge of diagnosing PHA, since PHA lacks specific characteristics in imaging that differentiates it from other hepatic lesions. On the other hand, biopsy in such a well-vascularized tumor is associated with a considerable risk for bleeding or tumor rupture, and can be misleading if the probe is not taken from vital tumor parts. Besides that, there is no clear and consistent histological classification for PHA, even after surgical resection [5,6,7].

Cytotoxic chemotherapy for PHA, in adjuvant as well as palliative settings, is another treatment option, and the results have been demonstrated before. Doxorubicin-based regimens and taxanes are considered effective, and are currently often used for first- or second-line therapy [19,20]. However, neoadjuvant therapy concepts have not been implemented for PHA yet, and robust studies in this respect in the future are less probable due to the low number of cases. In our cohort, none of the patients received neoadjuvant therapy, but four patients (44.4%) received adjuvant chemotherapy. Due to the small sample size, we found no significant difference in OS. Hence, radical resection may probably remain the primary recommended treatment for PHA.

The outcome of other malignant hepatic lesions (e.g., colorectal liver metastases) has improved significantly due to multimodal therapy approaches and technical advances [21,22]. In particular, the implementation of minimally invasive liver resection has led to a better postoperative outcome for patients, without jeopardizing the oncological outcome [23,24,25]. Additionally, other approaches such as tumor embolization, ablation, and portal vein embolization (PVE), have increased the eligibility of patients for liver resection for other hepatic malignancies [26,27,28]. These recent advances may improve the oncologic outcomes for PHA as well. The utilization of transarterial embolization (TAE) and transcatheter arterial chemoembolization (TACE) in PHA have been reported before, but only in a very small numbers of patients, and the comparison of long-term outcomes between those treatments is absent in the literature [15,29]. Nevertheless, due to persisting poor outcomes of hepatectomy for PHA over the decades in several reports, we see an urgent need for strategy adjustment for PHA treatment. While emergency embolization is an alternative option to surgery in ruptured cases, Pierce et al. demonstrated a median survival of 19 days for seven PHA patients treated by embolization, which was the worst among all entities in their cohort [30]. On the other hand, elective embolization alone, or embolization followed by surgery, seems to provide promising results for this entity [15,19,29,30]. The value of ablation therapy as a primary treatment for PHA is limited by the fact that most patients present with lesions larger than 5 cm. However, it could be an alternative option for smaller lesions [31].

The current study includes a comparably large number of resected PHA cases. We aimed to perform a meta-analysis of comparable studies that included more than five patients surgically treated for PHA. However, only four previous works were eligible for this comparison (Table 3). This shows the urgent need to further data on the topic, and the value of the current study. Nevertheless, our retrospective study was still confined by a small number of patients due to the rarity of PHA. The ability to detect small differences between groups and to adjust for potential confounders is limited. It remains difficult to deduct advice for treatment strategies based on a total number of nine patients.

Few attempts to establish national registers for PHA cases have been made so far, such as the British hepatic angiosarcoma register [32]. In our opinion, only further multicentric analyses and national, or even international, databases for PHA will help provide further understanding of this disease. Martínez et al. recently demonstrated this by collecting data from 18 US registries and analyzing the outcome of 366 patients with PHA. In their study, only 52 (14.2%) were surgically treated and had a median OS of 8 months, which is comparable to our results [33].

**Table 3:** Comparable studies of surgically treated PHA.

Author	Year	Number of Cases	R0 (%)	OS (Months)	OS for R0 (Months)
Matthaei et al. [5]	2009	22	82	30	39
Zhou et al. [18]	2010	6	83	12	14
Tripke et al. [14]	2019	9	88	18	59
Martinez et al. [33]	2021	52	n.a.	8	n.a.
Present study	2022	9	66.7	5	15

OS, overall survival; n.a., not available.

## 5. Conclusions

Our results for this bicentric study correspond to the current literature on PHA, and confirm the poor prognosis of the disease, even after surgical treatment. While radical resection is significantly associated with better OS, postoperative morbidity is still very high. Patients requiring emergency surgery due to tumor rupture might be challenging to resect radically, and may therefore have a high mortality rate. Multimodal treatment strategies, including surgery, systemic therapy, and interventional techniques, may deliver more satisfying outcomes in the future.

## Figures and Tables

**Table 1:** Demographic data of patients.

Characteristics	Surgically Treated Angiosarcoma (*n* = 9)	Notes	*p* Value ^a^
Age	72 (44–82)		
Gender (female)	5 (55.6)		0.73
ASA			0.73
≤2	5 (55.6%)		
>2	4 (44.4%)		
BMI	27.2 (20.0–39.1)		
Clinical presentation		In asymptomatic patients, lesion was found incidentally on imaging	
No symptoms	3 (33.3%)		
Upper abdomen pain	4 (44.4%)		
Tumor rupture	2 (22.2%)		
Liver cirrhosis	2 (22.2%)		0.09
Number of lesions			0.09
Solitary	7 (77.8%)		
>1	2 (22.2%)		
Size of biggest lesion	9.4 (3.4–18.5)		
Biggest lesion > 5 cm	7 (77.8%)		0.09
Tumor markers		Normal range	
AFP	4.1 ± 2.1	<7.0 ng/mL	
CEA	1.2 ± 0.4	<5 ng/mL	
CA 19-9	30.3 ± 18.6	<27 U/mL	
Lesion biopsy performed	6 (66.7%)		
Lesion biopsy confirmed PHA	4/6 (66.7%)		
Liver resection			0.73
Minor	5 (55.6)		
Major	4 (44.4%)		
Procedure			0.09
Open	7 (77.8%)		
Laparoscopic	2 (22.2%)		
R0	6 (66.7%)		0.31
Lymphadenectomy	5 (55.6)	N status not described in any patient	0.73
Blood Transfusion	6 (66.7%)		0.31
Length of surgery (min)	198.4 (124–255)		
ICU (days)	5.3 (2–23)		
ICU readmission	1 (11.1%)		0.02
Reoperation	1 (11.1%)		0.02
Morbidity	8 (88.8%)		0.02
30-day mortality	2 (22.2%)		0.09
90-day mortality	4 (44.4%)		0.36
Clavien–Dindo			0.36
0	1 (11.1%)		
≤IIIa	4 (44.4%)		
>IIIa	4 (44.4%)		
Adjuvant chemotherapy	4 (44.4%)	Pacilitaxel ± Gemcitabin or Doxorubicin + Ifosfamid	0.31

ASA: American Society of Anesthesiologists, BMI: body mass index, AFP: alpha fetoprotein, CEA: carcinoembryonic antigen, CA 19-9: carbohydrate antigen 19-9, PHA: primary hepatic angiosarcoma, ICU: intensive care unit, ^a^: Fischer’s exact test.

**Table 2:** Cox regression of parameters on overall survival.

Variables	Univariate Analysis	Multivariate Analysis ^#^
	*p* Value	HR (95%CI) *p* Value
ASA, ≤2 vs. >2	0.42	
Sex, male vs. female	0.42	
Age, ≤60 years vs. >60 years	0.50	
Other lives diseases, yes vs. no	0.37	
Ruptured tumor, yes vs. no	0.005	NS
Number of metastases, solitary vs. multiple	0.72	
Size of biggest lesion, ≤5 cm vs. >5 cm	0.07	
Liver resection, minor vs. major	0.45	
Liver resection, open vs. laparoscopic	0.50	
Lymph node dissection, yes vs. no	0.34	
Adjuvant chemotherapy, yes vs. No	0.21	
R status, R0 vs. R1	0.04	NS
Blood transfusion, yes vs. No	0.77	
Clavien–Dindo, 0 vs. ≤3a vs. >3a	0.37	

^#^ Cox regression multivariate analysis included all variables with *p* < 0.05 in univariate analysis. CI, confidence interval; HR, hazard ratio; ASA, American Society of Anesthesiologists; NS, not significant.

## Data Availability

The data presented in this study are available on requent from the corresponding author. The data are not publicly available due to ethical obligation.
